# Bile acids mediated potential functional interaction between FXR and FATP5 in the regulation of Lipid Metabolism

**DOI:** 10.7150/ijbs.44774

**Published:** 2020-06-14

**Authors:** Anita Kumari, Dharam Pal Pathak, Shailendra Asthana

**Affiliations:** 1 Translational Health Science and Technology Institute (THSTI), Faridabad, Haryana, India.; 2 Delhi Institute of Pharmaceutical Sciences and Research (DIPSAR), New Delhi, India.; 3 Delhi Pharmaceutical Sciences and Research University (DPSRU), New Delhi, India.

**Keywords:** Fatty liver diseases, FXR, FATP5, Bile acids, *cyp7a1*, Triglycerides

## Abstract

Perturbation in lipid homeostasis is one of the major bottlenecks in metabolic diseases, especially Non-alcoholic Fatty Liver Disease (NAFLD), which has emerged as a leading global cause of chronic liver disease. The bile acids (BAs) and their derivatives exert a variety of metabolic effects through complex and intertwined pathways, thus becoming the attractive target for metabolic syndrome treatment. To modulate the lipid homeostasis, the role of BAs, turn out to be paramount as it is essential for the absorption, transport of dietary lipids, regulation of metabolic enzymes and transporters that are essential for lipid modulation, flux, and excretion. The synthesis and transport of BAs (conjugated and unconjugated) is chiefly controlled by nuclear receptors and the uptake of long-chain fatty acids (LCFA) and BA conjugation via transporters. Among them, from *in-vivo* studies, farnesoid X receptor (FXR) and liver-specific fatty acid transport protein 5 (FATP5) have shown convincing evidence for their key roles in lipid homeostasis and reversal of fatty liver disease substantially. BAs have a wider range of biological effects as they are identified as modulators for FXR and FATP5 both and therefore hold a significant promise for altering the lipid content in the treatment of a metabolic disorder. BAs also have received noteworthy interest in drug delivery research due to its peculiar physicochemical properties and biocompatibility. Here, we are highlighting the connecting possibility of BAs as an *agonist* for FXR and *antagonist* for FATP5, paving an avenue to target them for designing synthetic small molecules for lipid homeostasis.

## Introduction

**Figure 1:**
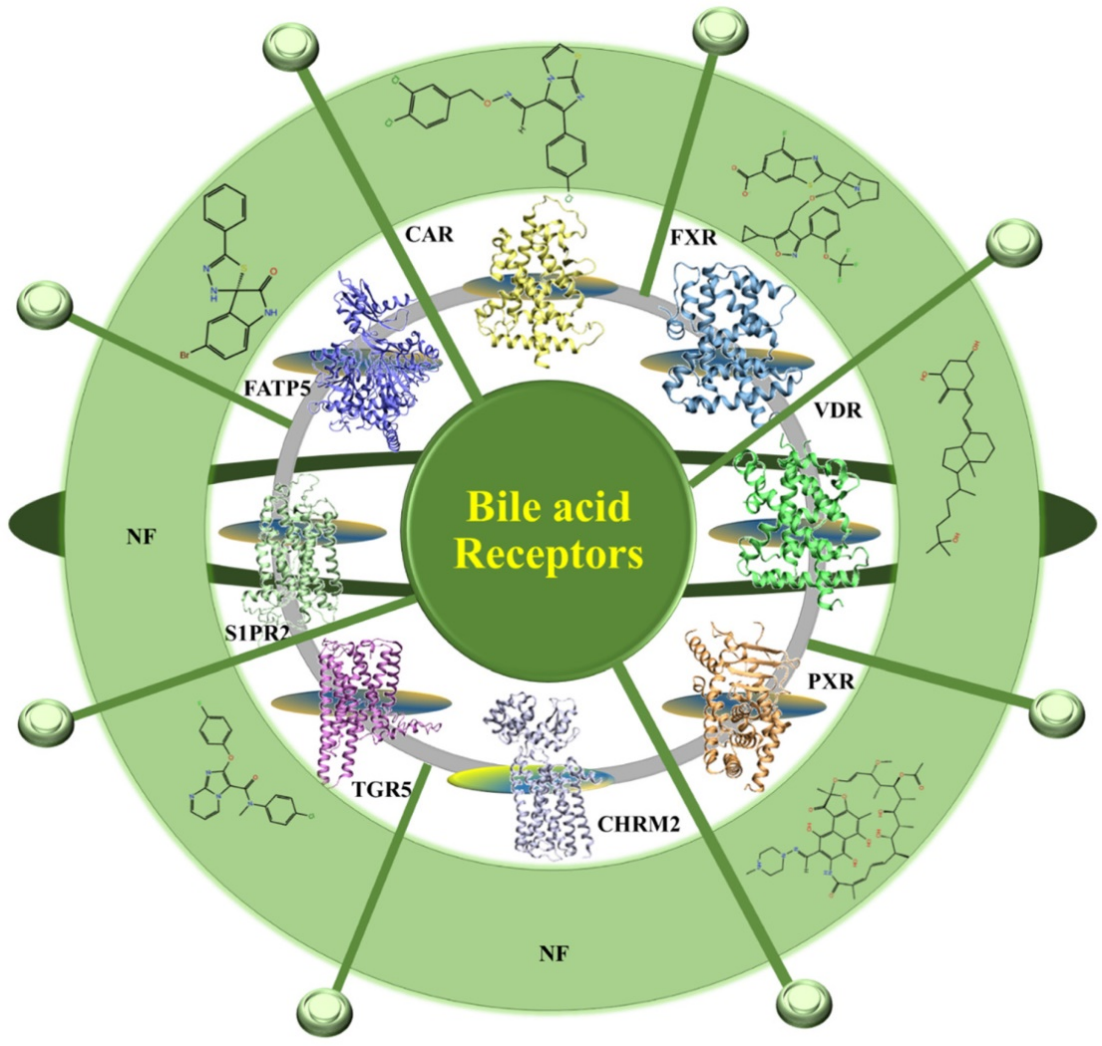
** Schematic overview of BA receptors**. The available crystal structures of BA receptors are FXR, PXR, VDR, CAR, CHMR2 are shown in 3D and in cartoon representation. The homology models of TGR5 and FATP5 were developed in the absence of their crystal structure. All the BA receptors are summarized in the Figure. The 2D structures of the available modulators of BAs receptors are shown (the small molecule which is reported against metabolic disorder are only picked). NF is not found.

The prevalence of the fatty liver disease in association with the epidemic of obesity and type 2 diabetes has increased worldwide and affects 15-40% of the general population 1,2. The fatty liver disease clusters with metabolic abnormalities, including type 2 diabetes, obesity, hypertension, hyperlipidemia which affects ~25% of the adult population worldwide 3,4.These metabolic abnormalities are linked to the alternation in the bile acid metabolism. BAs are endocrine molecules that contribute to several essential functions including cholesterol catabolism and intestinal lipid emulsification and, known for regulating the BA pool and lipid metabolism 3. The liver is the site for the BAs synthesis and converts cholesterol to BAs through the classic and alternative pathways 5,6. Here, the hepatic enzymes generate free primary BAs such as chenodeoxycholic acid (CDCA) and cholic acid (CA). In gut, the action of bacterial enzymes converts primary BAs into secondary BAs such as deoxycholic acid (DCA) and lithocholic acid (LCA). These primary as well as secondary BAs activate the nuclear receptors such as FXR, PXR (pregnane X receptor), VDR (vitamin D receptor), CAR (constitutive androstane receptor) and membrane-bound receptors including G protein-coupled receptor (TGR5), sphingosine-1-phosphate receptor 2 (S1PR2), cholinergic receptor muscarinic 2 (CHRM2) (7,8) (**Figure 1**) and various cell signaling pathways (c-jun N-terminal kinase 1/2, AKT, and ERK 1/2 (extracellular signal-regulated kinases)). The activation of these receptors alters the expression of various genes involved in the regulation of BAs, glucose, fatty acid, lipoprotein synthesis, metabolism, transport, and energy metabolism. In the liver, the BAs regulate the expression of a number of transport proteins and biosynthetic enzymes which are crucial for the maintenance of BA and lipid homeostasis 9. These include NTCP (Na+ dependent taurocholate cotransport peptide) 10, BSEP (bile salt export pump) 11 and *cyp7a* (Cholesterol 7 alpha-hydroxylase) which is the rate-limiting enzyme for the production of BAs from cholesterol via the neutral biosynthetic pathway in the liver 12. The function of these dedicated bile acid receptors has been well documented and studied before 7,13. In **Figure 1** we have given the available 3D structure for the receptors and their available synthetic molecules against them. Besides all this the liver also plays as a central organ for coordinating metabolism and has a large capacity for fatty acid (FA) uptake 14,15. Among these targets where expression and/or activity modulate lipid homeostasis, there is evidence of the BA-mediated link between FXR and FATP5 in the liver and lipid specific manner, which provide an idea about their involvement in NAFLD 16. It embraces two disease states i.e. Non-alcoholic Fatty Liver (NAFL) which is a broad, mostly benign liver disease and Non-alcoholic steatohepatitis (NASH) an inflammatory and progressive condition. For the first time, Stahl *et.al.* reported that the BAs modulates the FXR and FATP5, respectively, which is considered to have a large impact on metabolic disorders, especially those related to the metabolic syndrome 17. These two targets majorly modulate the lipid homeostasis in the liver 15,17-19. Therefore, it is demanding to explore the functional and biological link between BA-FXR-FATP5 to rationally mimic the endogenous BA for the discovery of small synthetic compounds that can narrow down the lipid uptake as an *antagonist* of FATP5 and enhance liver health and resensitize the BA pool substantially as FXR *agonist* in pathological condition.

## Structural Architecture at Atomic Level and elucidation of possible Molecular Mechanism can pave a path for Lead Discovery

In the current scenario the cornerstone of modern medicinal chemistry is ligand- and Structure-based drug discovery (LB/SBDD) which has inspired numerous small molecule lead optimization efforts and play a role in the discovery of drugs and drug like molecules immensely 20,21. The protein structures have increased exponentially and now confine highly dynamic targets also which was previously impervious to crystallography. X-ray and NMR structures of proteins (APOs) and co-crystals with ligand complexes (HOLOs) are generally available and provide a platform for structure-guided drug discovery as the structures are typical of sufficient resolution at the atomic-level to stimulate effective hit identification and design and their conversion from identified hit-to-lead molecules i.e. lead optimization 22. However, the interaction maps of frozen state of protein-ligand observed in X-ray structures are in general not good enough to explain the structure-activity relationships (SAR) well and this can lead to inaccurate and futile synthesis cycles. Nevertheless, molecular dynamics simulation appears a strong tool to understand the dynamical changes of different states of proteins such as transition state, open-to-close state, conformational change states like recruitment of co-factors, prosthetic group, post-translational modifications, role of mutations, changes in the binding site architecture and allostery. Therefore, a curation of possible structural and ligands information seems to be a priority to explore the key targets for drug discovery.

### About FXR

**Figure 2:**
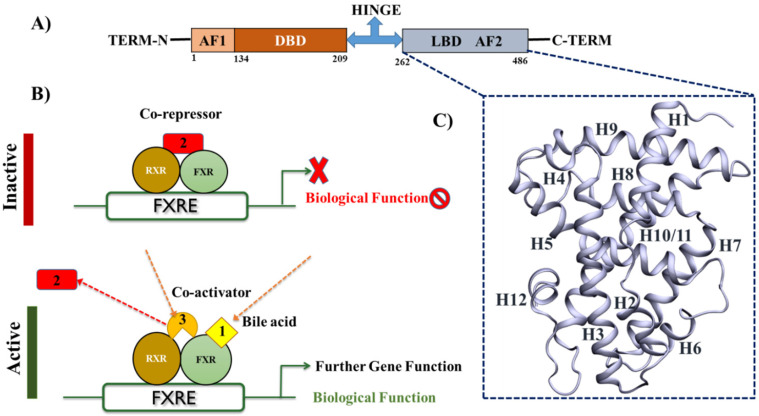
** Structural-functional organization of FXR A)** The schematic representation of the FXR domains structure **B)** FXR forms a heterodimer with retinoid X receptor (RXR) that binds to the FXR-response element (FXRE), a DNA sequence in the promoter of its target genes. In the absence of ligands, the FXR heterodimer is associated in complex with corepressor, leading to transcriptional repression. Following BAs binding to the LBD of FXR, the heterodimer undergoes conformational changes, leading to the recruitment of co-activators to replace corepressors, which results in the transactivation of target genes expression. **C)** The 3D structures of LBD of FXR by using the PDB-ID: 5Q0K.

FXR is a ligand-activated transcription factor, belongs to the NR (nuclear receptor) superfamily and is essential in regulating a network of genes involved in maintaining BA and lipid homeostasis. The molecular mechanism of FXR is extensively studied; however, limited information is available about FATP5 and its modulation. FXR exerts its function by eliciting transcriptional alterations 23. It binds to DNA as a heterodimer with RXR (retinoid X receptor) or as a monomer to regulate the expression of various genes. In common with other NRs, the FXR protein exhibits a modular structure, and contain several autonomous functional domains: N-terminal region with a ligand-independent activation function (AF1), a highly conserved zinc-finger DNA-binding domain (DBD) that is connected to the ligand-binding domain (LBD) by a flexible hinge region (**Figure 2A**) 24. Upon *agonist* binding to FXR creates the huge conformational rearrangement due to which the dissociation of co-repressors and the recruitment of co-activators occur to promote the transcriptional initiation (**Figure 2B**). The LBD contains two well-conserved regions: a signature motif and the AF2 motif located at the carboxy-terminal end of the domain, which is responsible for the ligand-dependent transactivation function (**Figure 2C**).

**Figure 3:**
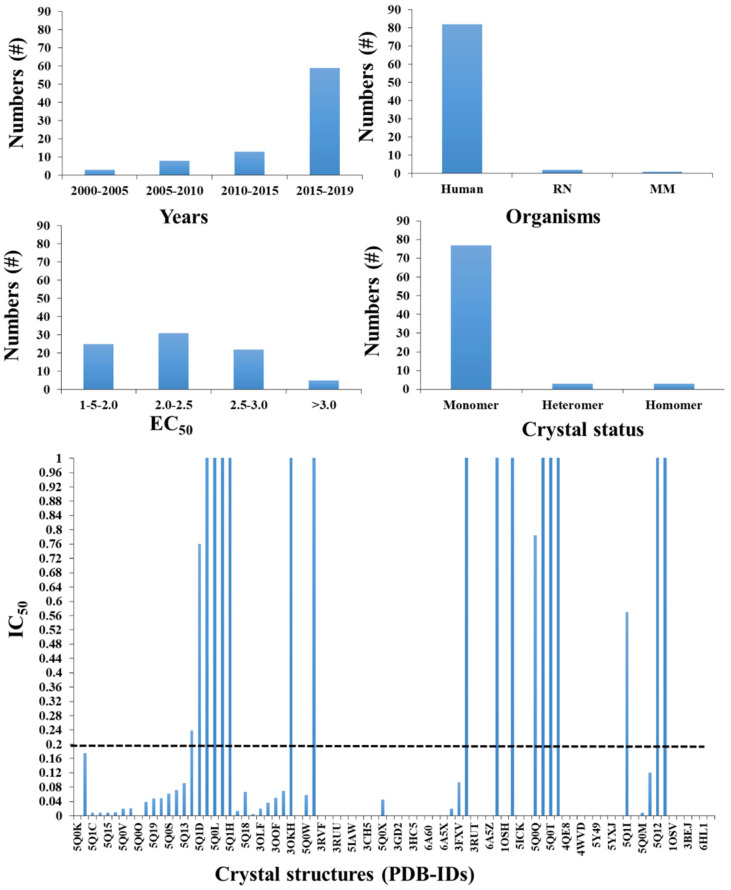
** The available crystal structure for FXR (different species) with their biological activity and their existing form with RXR**: A) the total number of FXR crystals published year-wise, B) number of organisms, C) The co-crystal structures with reported *agonist* and *antagonist* based on their EC50, D) the type of FXR crystal reported and E) the best compounds (below 1uM) is only shown here. The dotted line is used as a cutoff for low micromolar compounds for a ligand-based approach.

To date, 84 FXR structures have been reported to the Protein Data Bank (PDB), which was up to ~25 in number till 2015, indicating the importance of FXR study in the current scenario **(Figure 3)**. Considerable interest in drug discovery and pharmacology in the recent past is due to its important role in metabolism and its value as a drug target to treat liver disorders and metabolic diseases. The details of the available crystal structures of FXR with their available biological activity are reported in **Figure 3**. Despite all these available crystal structures, there are several areas which need structural explanations such as flexibility of FXR, which reveals clearly from different crystals/co-crystals about its structural conformations. The activity data shows that there are molecules which are having potent IC_50_ and EC_50_ values (**Figure 3**).

This information possibly provides a rational basis for FXR structure- and ligand-based drug design which has enormous potential to yield a novel molecule with optimal selectivity, potency, and efficacy profiles. It is also addressable whether FXR agonism is required and/or partial-agonism is good enough for lipid modulation in a diseased condition. Recently, it is reported that the pharmacological administration of full FXR-*agonist* has been plagued by mechanism-based side effects 25. Since NR is known to participate in various endocrine functions, FXR modulation is prone to cause mechanism-based side-effects. Clinical trials of OCA have already shown that extensive FXR activation disrupts cholesterol homeostasis with FXR activation blocking the metabolic conversion of cholesterol to BAs via SHP/FGF19 up- and *cyp7a1* down-regulation. Since this pathway constitutes the main route of metabolic cholesterol elimination, its long-term pharmacological blockade may have serious consequences. Partial activation appears to be an avenue to safely exploit FXR as a drug target as it could reduce side effects such as loss of metabolic cholesterol degradation. Similar strategies have been proposed for other BAs sensors that are known for causing unwanted effects. However, to explore this strategy, a better understanding of partial FXR activation on a molecular level is necessary. The comparison of FXR-LBD co-crystal structures in complex with *agonists*, *antagonists*, and *partial agonists* revealed several significant differences and these differences seem to affect the conformation of the FXR-LBD explaining different pharmacological effects.

From the structure point of view, it is essential to know about key residue and structural determinants for partial agonism, the positions of H12 and the co-activator/co-repressor bound to the FXR-LBD and loss/gain of interaction networks (like hydrogen bonds, VdW interaction, and non-bonded contacts, etc.), as it has been seen that recruitment of co-activator/co-repressor itself inducing the significant conformational changes. Since a co-crystal structure provides only a single snapshot of dynamic binding equilibrium and here, it is not sufficient to gain mechanistic understanding.

From structures, it is well evidential that loops between (H1-H2, H5-H6, and H6- H7) and helices H11 and H12 are the key determinants for the recruitment of *agonists*, *partial-agonist* and *antagonist*, and their biological function. NMR and x-ray crystallography provides enough clues about structural and conformational variations of FXR with different interacting partners, co-activators and co-repressors. Since NMR has its own limitations and crystal structures are static in nature, a multidisciplinary approach is required to unravel the molecular changes in the FXR structure that may lead to the discovery of novel selective FXR modulators. A better understanding of FXR's molecular mechanism may significantly support future drug discovery of safer, optimal selectivity, potency and effective FXR modulating agents 25.

### About FATP5

**Figure 4:**
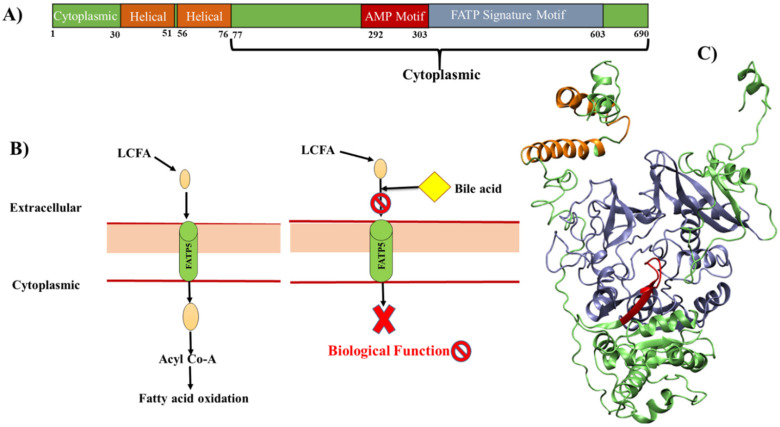
** A model for cellular fatty acid uptake**: **A)** The domain structures of FATP5 receptor, **B)** extracellular LCFAs bind directly to FATP5 and be transported into cells. Intracellular LCFAs would then be coupled to CoA and further got oxidized. The BAs bind the extracellular domain of FATP5 and inhibit the uptake of fatty acids. **C)** 3D model is generated based on homology and fragment-based modeling approach. The FATP5 is rendered in cartoon and the color code is the same as shown in panel (A).

The FATPs comprise a family of 6 members, which encode FATP1-6 26. Among all the family members, mainly FATP5 has been known to exert tissue-specific effects in regulating BA synthesis and LCFAs transport and can reduce the lipid accumulation; therefore, it appears as a potential target for rational drug design 17. FATPs are integral membrane proteins, ranging from 63-80 kilo Daltons (kDa) in size with the transmembrane domain. The N-terminus and C-terminus are located on the extracellular/luminal side and on the cytosolic side respectively (**Figure 4A**) 27,28. All FATPs members are characterized by the presence of a highly conserved, 311-amino acid signature sequence known as the FATP sequence, as well as an AMP binding domain (292-303), located on the C-terminus (**Figure 4A**). This region is responsible for the binding and uptake of LCFA and is commonly found in members of the ACSL family 29-31. The cellular capacity of LCFA uptake depends on transporter proteins and BA acts on the extracellular domain of FATP5 as competitive inhibitors (**Figure 4B**), further studies are needed to explore the mechanism of BAs on FATP5 17.

Fatty acid uptake factors such as CD36, FATP2, and FATP5 actively function when excessive fatty acids are present owing to high fat intake or obesity, but not in normal conditions. It is also studied that there is a decrease in hepatic FATP5 expression in NAFLD that is associated with the hepatic fat loss during NASH progression to cirrhosis 32. The role of FATP5 as a tumor suppressor in HCC (Hepatocellular carcinoma), both *in vivo* and* in vitro*, has been studied and provides a mechanistic link between disrupted lipid metabolism and redox homeostasis 33. From the structural point of view, there is no crystal structure available till now. But the helical and AMP binding sites possibly appear as the modulating sites in FATP5 either for BA's mimics and/or for small molecules (**Figure 4**). There are few molecules known for FATP5 as compared to FXR, but there are secondary bile acids (DCA and UDCA) which act on FATP5 and ability to inhibit the TG accumulation 17. There are also some preliminary findings which showed that the bile acid (chenodiol and ursodiol) selectively target the FATP5 by employing the HTS (high throughput screening) assay 34. The structure and their details we have discussed further in the paper. This structural based knowledge of FATP5 protein would help the discovery of new drug leads against metabolic disorder.

## Tissue-specific role of FXR and FATP5 in lipid metabolism

### In Liver

**Figure 5:**
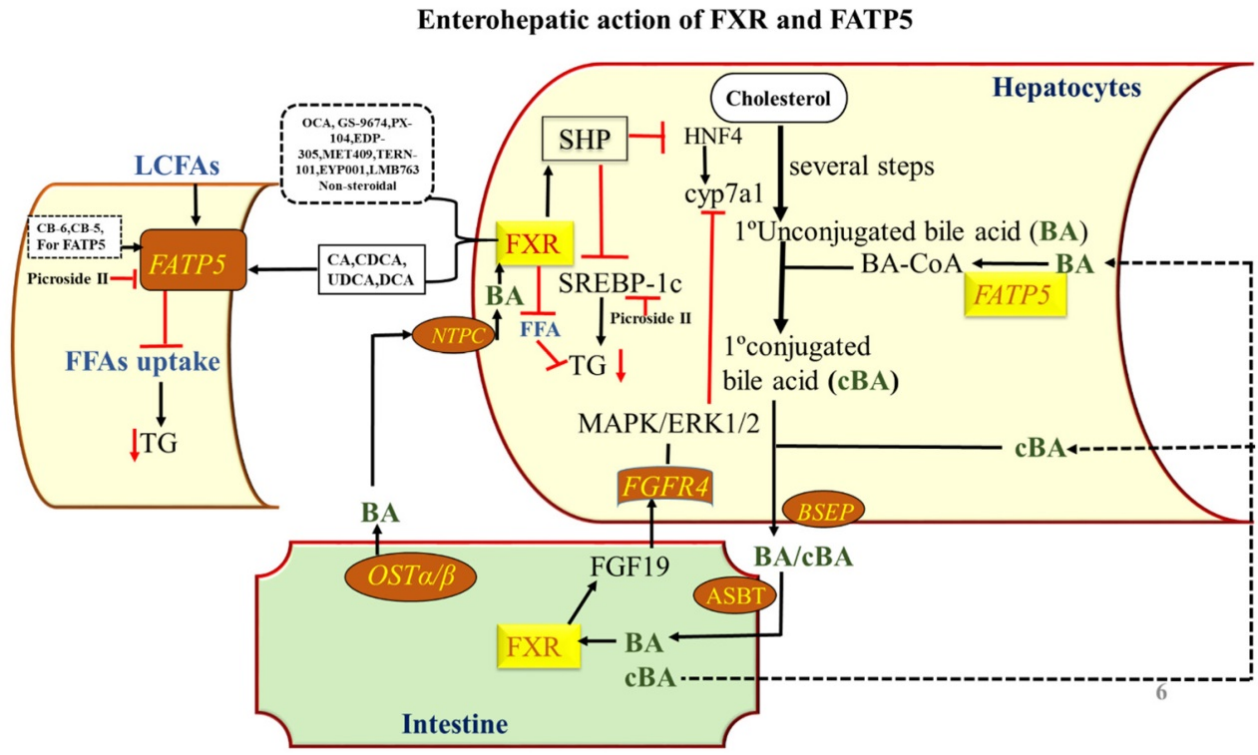
** Enterohepatic action of FXR and FATP5.** Role of FXR and FATP5 in synthesis, transport, and enterohepatic circulation of BAs and the effects of steroidal and non-steroidal *agonists* on the activity of the same. In the liver, activation of FXR by *agonist*s induces SHP to inhibit *cyp7a1* gene transcription that further allows the liver to downregulate the BA synthesis in response to maintaining a constant BA pool. The TG level is reduced by SHP acting on *SREBP-1c*. BAs are secreted via BSEP into the gallbladder and reabsorbed via ASBT in terminal ileum enterocytes. Here, they bind and activate FXR, which stimulates production and secretion of FGF15/19 into the portal circulation. BAs activate FXR in the intestine to induce FGF15/19 which is transported to hepatocytes to activate FGFR4, which further activates a signaling pathway involving MAP kinases and causes repression of *cyp7a1* transcription, thus downregulating BA synthesis. After this the OSTα/β-mediated secretion into the portal circulation, BAs are taken up by the liver via NTCP, completing the enterohepatic cycle. The FATP5 involved in the uptake of fatty acids and the conjugation of BAs in the liver. The FATP5 conjugates the BA by BA-CoA enzymatic activity. The BAs inhibit the uptake of long-chain fatty acid in FATP5 dependent manner and inhibits the TG level in the liver and maintain the lipid homeostasis in the liver. The endogenous ligand and the synthetic ligand for both the receptors are given in the figure.

**Figure 6:**
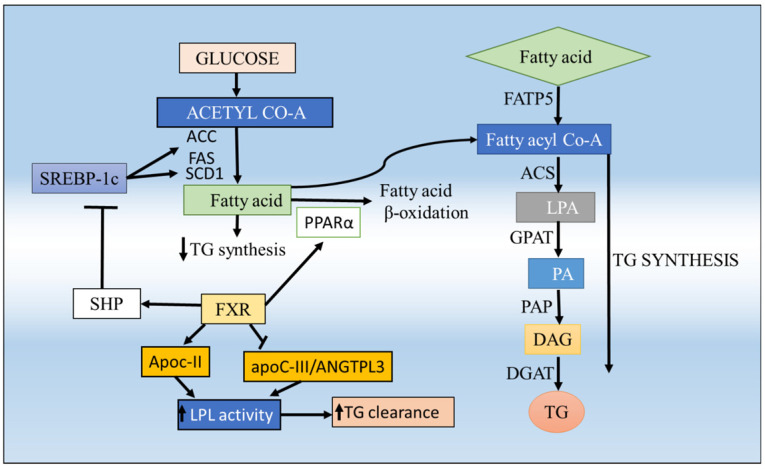
** Regulation of lipid homeostasis by hepatic FXR and FATP5 and their involvement in hepatic steatosis**. FFA can be synthesized de novo from glucose, mainly regulated by SREBP-1c. Activation of hepatic FXR lowers plasma FFA and TG, resulting from (i) repression of hepatic TG and fatty acid (FA) synthesis as a result of SHP-dependent inhibition of SREBP-1c; (ii) induction of ApoC-II and repression of ApoC-III and ANGTPL3 in the liver, resulting in enhanced lipoprotein lipase activity; thus promoting clearance of TG-rich lipoproteins and (iv) induction of human PPARα and FA β-oxidation. The FATP5 plays an important role in fatty acid uptake to the liver upon a high-fat diet which contributes to the development and/or aggravation of fatty liver. Consequently, the incorporation of fatty acyl CoA into TG is greatly enhanced and lipid accumulation occurs in the liver.

Selective targeting of FXR and FATP5 in specific tissues seems a promising strategy to increase the therapeutic index of their modulators and reduce the side effects compared to whole-body targeting. The role of nuclear receptors in BA metabolism is well explained by Li and Chiang *et.al.* 5, where in the liver, FXR is a key sensor of BA and has a central role in maintaining BA homeostasis and to protect liver cells from potential deleterious consequences of cellular BA overload and therefore it becomes an attractive target for the metabolic disorder 19. FXR is dominantly expressed in enterohepatic system; however, it is also expressed in other tissues including adrenal glands, kidney, stomach, heart, and macrophages 35 as well as in white and brown adipose depots 36,37, that's why by restricting the tissue-specific activity. In enterohepatic circulation of BAs**,** FXR plays the key mediator role in the BA feedback repression mechanism by primarily inducing the expression of small heterodimer partner (SHP), which further inhibit transcription of the *cyp7a1* gene that allows the liver to downregulate the BA synthesis in response to an increase in BA levels and thus maintains a constant BA pool 38,39 (**Figure 5**). It also works as an important component of lipid homeostasis, most likely in the regulation of enzymes and transporters that are critical for lipid catabolism and excretion 9. Upon BA activation of FXR induces BSEP (bile salt export protein) 40 and MRP2 (multidrug resistance-associated protein 2) in hepatocytes 41 (**Figure 5**). On the other hand, FXR also repressed the NTCP as a feedback inhibition of BA uptake to prevent liver injury 42. The studies of FXR null mice have revealed that the reduced capacity to excrete BAs and the level of hepatic TGs also significantly greater than that for wild-type mice, which provides convincing evidence for a central role of FXR in BAs homeostasis 9. Activation of FXR inhibits *cyp7a1* and reduces BA synthesis, and inhibits NTCP and OATPs to reduce sinusoidal uptake of BAs to maintain the BAs homeostasis 9. There are a number of in-vitro and in-vivo studies using mouse models for FXR that have elucidated the positive relationship with fatty liver disease and its effect on the regulation of lipid metabolism 9,43-45. The study of FXR -/- mice confirmed that it was critically involved in lipid homeostasis through regulating the cholesterol catabolism, transport, and lipoprotein metabolism 9. FXR suppresses sterol regulatory element-binding protein-1c (*SREBP-1c*), reduces the hepatic triglyceride (TG) level (**Figure 5**). The treatment of mice with FXR agonists results in the repression of *SREBP-1c*. So *SREBP-1c* functions as a critical transcription factor that regulates many genes involved in both fatty acid and TG synthesis 46. The activation of FXR also represses hepatic de novo lipogenesis and stimulates fatty acid β-oxidation by inducing expression of Peroxisome proliferator-activated receptor -alpha (PPARα), limiting hepatic lipid accumulation 47-49 can also induce the expression of ApoCII (apolipoprotein CII), an activator of lipoprotein lipase, so that promote plasma very-low-density lipoprotein TG clearance and suppressing the expression of ApoCIII (apolipoprotein CIII), an inhibitor of lipoprotein lipase activity 50,51. Altogether, these data suggest that FXR activation lowers plasma TG levels via both repressing hepatic lipogenesis and TG secretion, and increasing the clearance of TG-rich lipoproteins from the blood (**Figure 6**). These studies suggesting the expression level of FXR also acts as a determinant of lipid metabolism and has been suggested as a promising therapeutic target for hepatic metabolic disorders 52.

The study also shows that the lipid homeostasis, in general, is largely a protein-mediated process requiring FATP5 and helps in the understanding of the liver function and disease 53. Disturbed fatty acid metabolism is one of the causes involved in the pathogenesis of NASH 54. The uptake of circulating fatty acids to the liver is largely dependent on fatty acid transporter protein (FATP), CD36/fatty acid translocase (CD36/FAT) and fatty acid-binding protein-plasma membrane-bound (FABPpm) which play an important role in the trafficking of the FFA 55,56. Among these, the FATP5 is widely expressed in liver and localized to the basal plasma membrane of the hepatocytes 53 and the deletion of FATP5 influence the development of hepatic steatosis 53 but in CD36 it does not show the same effect 57 which clearly indicates the important role of FATP5 over other transporters. Consequently, the incorporation of fatty acyl CoA into TG is greatly enhanced and the machinery of lipid accumulation occurs in the liver (**Figure 5**). In humans, the FATPs comprise a family of 6 members that contain a common motif for fatty acid uptake and fatty Acyl-CoA synthetase function 58. All these transporters are helping in the uptake of FAs in the multiple body tissues like kidney, skeleton muscles, heart, and adipose tissues furthermore the oxidation of FA occurs. It mainly affects the liver where the FATP5 protein plays an important role in the uptake of fatty acid. The detailed analysis of the hepatic FATP5 knockout in livers shows that the alternation in the lipid homeostasis which supports the hypothesis that efficient hepatocellular uptake of LCFAs, and thus liver lipid homeostasis in general, is largely a protein-mediated process requiring FATP5. These new insights into the physiological role of FATP5 should lead to an improved understanding of liver function and disease 53. The altered fatty acid metabolism is a hallmark of numerous metabolic diseases and pathological conditions such as NAFLD, which is linked to obesity and types 2 diabetes and insulin resistance 59. So the fatty acid transporters can be used as a target to rectify lipid fluxes in the human body, specifically in the liver and regain metabolic homeostasis. The knockdown studies of FATP5 is able to reverse NAFLD, results in considerably improved glucose homeostasis in an animal model (fed HFD), therefore, proposed critical for the sustained caloric uptake and fatty acid flux into the liver 59. Inhibition of uptake of FATP5 mediated fatty acids in the liver seems to be a potential avenue for the treatment of NAFLD. FATP5 presence is liver-specific and its knockout study reported that there is a significant drop (~50%) of lipid uptake which suggests that FATP5 contributes significantly to fatty acid uptake in primary hepatocytes 53. Moreover, it also has shown that it perturbed sugar homeostasis 59. The synthetic molecules which can block the cellular uptake of LCFA could be a key to identifying potential therapies for metabolic diseases. Therefore, targeting the FATP5 can rectify the lipid fluxes in the human body, specifically in the liver and regain metabolic homeostasis. Therefore, selective targeting of receptors in specific tissues also seems a promising strategy to increase the therapeutic index of modulators. The previous studies also report that the FATP5 also exhibits the bile acid CoA synthetase (BACS) activity, hence, essential for proper BA conjugation and involved in lipid metabolism 60. The gene expression analyses of the BA-CoA ligase FATP5 revealed that there is impaired BA amidation due to the decreased expression of this 61. The knockout and knockdown mice models for FATP5 also confirm that it is responsible for the majority of BACS in the liver and show a dramatic increase in unconjugated BAs which is suggesting that it plays a major role during reconjugation of BAs in the enterohepatic recirculation 18,60 (**Figure 5**). It was also investigated the severe conjugation defect in FATP5 knockout mice in contrast to wild-type mice, where 95% of BAs are conjugated, whereas only 17% of BAs are conjugated in FATP5 deletion mice 60. Moreover, in FATP5 knockout mice, there is an up-regulation of the SHP (**Table 1**) which mediates the inhibition of *cyp7a1* expression in response to high BA concentration although this regulation did not reach statistical significance. The FATP5 knockdown also resulted in a significant increase in genes involved in hepatic cholesterol biosynthesis (*SREBP2* pathway) and fatty acid synthesis (*SREBP-1* pathway) 18. The deletion of FATP5 also causes protection from obesity and hepatic TG accumulation and improved insulin sensitivity 3 and a significant reduction in lipid uptake (**Figure 6**). It was also examined in KO mice the total diglyceride and TG content was reduced by 59% and the LCFA uptake by 50% by using the gas chromatography/mass spectrometry-based analyses and FACS-based measurements, respectively 53. The loss of FATP5 causes the redistribution of dietary lipids away from the liver to other FFA metabolizing tissues. The *in-vivo* study for these receptors has shown the up and down-regulation of the target genes which involves the BA synthesis and uptake of fatty acids (**Table 1**) (**Figure 5**). The data suggest that the alterations in gene expression have a role in BA and lipid metabolism. Overall, FATP5 is a protein with multiple activities and known to play an essential role in the BA reactivation, hepatic FA uptake, and lipid accumulation. Henceforth, from all these observations, we found that gene expression level gets affected differently in FXR and FATP5.

### In Intestine

In the intestine the BAs are actively reabsorbed by the Apical Sodium-dependent Bile Acid Transporter (ASBT). FXR modulation provides significantly increased therapeutic benefits. FXR signaling provides cross-talk between the intestine and the liver via activating the expression of the enterokinase FGF15/19 (**Figure 5**). Activation FXR in intestine increase FGF15/19 expression, which further activates FGFR4, causes repression of *cyp7a1* transcription by signaling pathway involving the MAP kinase (A mitogen-activated protein kinase), thereby reducing hepatic BAs overload 62 (**Figure 5**). Constitutively active intestinal FXR improved BA homeostasis and reduced cellular proliferation, hepatic inflammation, and fibrosis in young FXR null mice 62,63 The therapeutic indications for the use of intestinal-specific FXR modulators may also be extended to liver disorders. In the intestine the BSEP and NTCP are up-regulated in FATP5 deleted mice (**Table 1**) which possibly allows increasing the transport of the remaining conjugated BAs 60. These tissue-specific roles of FATP5 and FXR suggest that both play a role in BAs metabolism and lipid homeostasis. These new insights into the physiological role of FATP5 and FXR might lead to an improved understanding of liver function and disease. With an in-depth understanding of FXR and FATP5 function and regulation at the cell-, gene- and tissue-specific levels, we will have more tools and be more confident in designing FXR modulators in the future to prevent and/or treat BA and lipid-related abnormalities in humans. Henceforth, the reduction of hepatic FATP5 through different routes such as via gene therapy or small molecular inhibitors is a novel tool to dynamically redirect lipid fluxes and may provide novel approaches for lipid homeostasis. These provide new insights into the physiological role of FATP5 which is poorly characterized as a drug target.

## Specific bile acid as endogenous modulators of FXR and FATP5

There are numerous pharmacological FXR modulators including *agonists* and *antagonists*, which have demonstrated that FXR plays a central role in controlling lipid homeostasis by reducing plasma TG and HDL (high-density lipoproteins) levels. The obeticholic acid (OCA) is ~100-fold more *agonistic* activity than the endogenous ligands chenodeoxycholic acid (CDCA) 64 and many *agonists* have been tested in clinical trials for treating the metabolic disorder 65 (**Table 2**). The steroidal (BA and their derivative) and non-steroidal (synthetic) *agonist* and *antagonist* are well known for the BA receptors as discussed in **Table 2** and have the modulation action on FXR and FATP5. The activation of FXR with BAs or synthetic activators has shown the reduced secretion of TG level in the liver and maintaining lipid homeostasis. The order of potency of bile BAs activating FXR is chenodeoxycholic acid (CDCA) > lithocholic acid (LCA) = deoxycholic acid (DCA) > cholic acid (CA), whereas one of the secondary BAs, LCA (lithocholic acid), is the most potent activator of TGR5, PXR and VDR 5. Among BAs, UDCA cannot activate FXR 66 and has no suppressive effect on *cyp7a1* in human hepatocytes up to ≤100 μmol/L 67. It is well documented that the BA inhibits FATP5 and reduces the TG level in *in-vivo* and *in-vitro* studies 17. The order of potency of BAs inhibiting FATP5 is: deoxycholic acid (DCA) > ursodeoxycholic acid (UDCA) > chenodeoxycholic acid (CDCA) > cholic acid (CA) 17. It is also noticeable that the BA like CA, CDCA, and DCA activate FXR but the UDCA was found to act as an FXR *antagonist* rather than agonist, having effects on BA and lipid metabolism in morbid obesity 68. The patients with NAFLD have shown the expression of FXR is less, which is associated with hepatic TG accumulation and hepatic steatosis 43. The available *agonist* and *antagonist* can alter the bile- and lipid- acid metabolism by acting on FXR at the same time BA can inhibit the uptake of fatty acid by acting on FATP5*.* The available data also suggests that the association of FATP5 in the pathogenesis of metabolic syndrome and steatosis 69. Interestingly, other than the BAs, Picroside II attenuated FFA accumulation in HepG2 (human liver cancer cell line) cells via downregulation of FATP5, *SREBP-1c* decreasing FFAs uptake and lipid synthesis 70 (**Figure 5**). Henceforth, to mimic the BAs which can work as an *agonist* for FXR and *antagonist* for FATP5.

## Bile acid a link between FXR and FATP5 for small molecule modulators

BAs are not the only function as physiological detergents to aid digestion of lipid nutrients but also function as signaling molecules that profoundly impact metabolism by activating nuclear and membrane BA receptors 13 and also by mediating induction of liver-specific FATP5 17. In excess amounts, BAs are toxic to the cell; therefore, their levels must be tightly controlled for the homeostasis of the cell. The nuclear receptor FXR and transporter protein FATP5 plays a key role in this regulation. Despite recent advances, the mechanisms underlying the interplay between the liver and intestine mediated by BAs-FXR and FATP5 to regulate BA levels, in particular, hepatic expression of *cyp7a1,* the rate-limiting BA synthetic enzyme, are not well understood. Studies have shown that BA can activate or suppress the FXR and well known for the treatment of metabolic disorder 71 Overexpression studies with hepatic FATP5 have shown that they not only enhance the LCFA uptake 53 but also activates the primary bile precursors 72. Here, we are exploring the BA-mediated link of FXR with FATP5 and a summary of the regulatory role of FXR and FATP5 in BAs transport, biosynthesis, and fatty acid uptake is presented in **Figure 5**. It is well documented that endogenous BAs like UDCA and DCA interferes with protein-mediated hepatic LCFA uptake and reduces the TG level up to 50% 17, and it is also well mounted that FXR and FATP5 are modulated significantly with BA 17,73. Thus these findings put forth a rationale to establish a link between hepatic FXR and hepatic FATP5 proteins. The studies suggest that FATP5 plays a role in hepatic TG metabolism via FXR 18. In the current scenario, there are few leads working as *agonists* against FXR 65, *antagonists* against FATP5 17, and few endogenous BAs are reported to work as a dual character such as *agonist* and *antagonist* both for FXR and FATP5, respectively 17,73. Therefore, there is a curiosity to unveil the functional and mechanistic biology to explore the molecular mechanism between FXR and FATP5.

## Curation of leads with their chemo types for ligand-based approach

**Figure 7:**
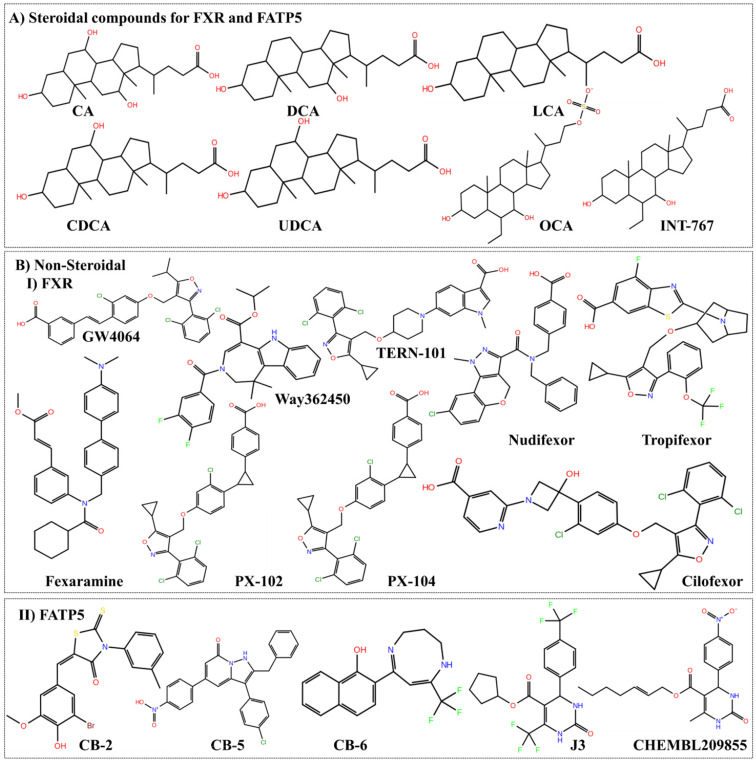
** The chemical structure of lead compounds. A)** The structure of BAs and their derivatives, which acts upon FXR and FATP5 both as highlighted in **Table 2**. **B-I) and B-II)** The non-steroidal compounds for FXR and FATP5, respectively. We here showed the molecules in advance phases for FXR. For FATP5 only those molecules are picked which have substantially potent and can be used for lead discovery.

From the known ligands chemical moiety, it would be possible for medicinal chemists to design and modify the possible chemical changes for better efficacy. The discovery of OCA is the best example of mimicking natural BAs. At present, several molecules for FXR evaluated in pre-clinical or clinical trials as* agonist, antagonist* and* partial agonist* as discussed in **Table 2***.* Indeed, there is no specific molecule for FATP5 that has been reported to date and few kinds of literature claim the BAs and its mimics possibly modulate the FATP5. The steroidal and non-steroidal ligands for FXR and FATP5 are shown in **Figure 7**. The availability of these compounds or the structurally related derivatives has contributed to characterize the receptor from a structural, pathophysiological and therapeutic point of view. This structural information may provide a room to optimize the compounds for their affinity, selectivity of receptor recognition and target gene modulation. These are the prerequisite for many of the future developments as FXR and FATP5 modulators.

## Conclusion

Extensive research over the last several decades has unveiled the several unrecognized functions of BAs which are mediated by activation of a group of its receptors and pathways. Here, we are trying to explore the possible insights for a targets like FATP5 which is less explored, indeed, it is a challenging target for drug ability but efforts required to crack it for the discovery of novel leads (especially non-steroidal and synthetic new chemical moieties) that selectively can target the metabolic pathways involved diseases and set the stage for the development of a novel generation of FXR and FATP5 targeting drugs with improved pharmacological actions. Therefore, it is required to unveil some specific properties of BAs relevant to their intrinsic potency and selectivity for particular receptors and to design novel modulators of these receptors with improved pharmacokinetic and pharmacodynamic profiles. In this regard, the structural, dynamical and functional understanding of established BAs which are known to be potent against FXR, must be tested against FATP5 also, in the pursuit of designing selective ligands that can regulate the lipid content by modulating these two targets.

## Future Perspective

BAs function in digestion and solubilization of lipophilic nutrients and as drugs in the small intestine, which are well documented. From the past two decades, there is emerging evidence which identified BAs as signaling molecules exerting the multiple physiological functions through complex and intertwined pathways that are largely mediated by modulation of different receptors as discussed above. There is extensive literature supporting the role of BAs in regulating metabolic processes. The role of tissue-specific FATP5 functions in the regulation of BA and NAFLD is emerging. BAs lower TGs level via a pathway involving FXR, SHP, and *SREBP-1c*. Basic research in BA metabolism and signaling suggest a scientific rationale for targeting the fatty liver diseases by inhibiting the FATP5 and activating the FXR. In this regard, we proposed that the BAs can modulate the FXR activity and inhibit the uptake of fatty acids through a protein-mediated process requiring FATP5, which appears promising for fatty acid modulation. These new insights into the physiological role of FATP5 should lead to an improved understanding of liver function and disease by modulating the close relationships of the BA and with the FXR. Although the signaling function of BAs and insights into the 3D structures (crystal structures) of BA receptors and their known ligands have accelerated the pace of discovery of new drugs for metabolic and liver disorders, limited has been explored for FATP5. The discovery and development of a therapeutic drug requires a more in-depth insight into the metabolic effects of each of the endogenous and synthetic BA, also it requires the pharmacological mechanism particularly at the molecular level on both FXR and FATP5. The clinical trial shows that the OCA disrupts cholesterol homeostasis by doing extensive FXR activation and blocking the metabolic conversion of cholesterol to BAs via SHP/FGF15/19 up- and *cyp7a1* down-regulation as discussed above. Also, there are some potent FATP5 inhibitors that are also reported, moreover, their effect has not been exploited for FXR and vice versa. In the current scenario, the industry and academia are more focused to design agonists to upregulate the target proteins to handle the diseased state similar to BA which is known for lipid homeostasis and its regulation. Since OCA found with several drawbacks like itching and elevated LDL levels and its marketed cost, also in pathological condition do we need *agonist* or *partial agonist*? or only the requirement of modulators that can resensitize the BA pool. Undoubtedly more pathways involved and more research needs to be done to understand the mechanism behind insulin resistance, an accumulation of hepatic lipids, initiation of hepatocyte apoptosis, an increase in inflammation, and an increase in extracellular matrix that eventually results in fibrosis, steatosis to steatohepatitis progression so that drugs that successfully treat NAFLD and NASH can be developed. Therefore, new targets involved in BA pathways must be explored by combining the arsenal of structure-based, ligand-based, medicinal chemistry and computational drug designing approaches to design the non-steroidal mimics of BA's as a polypharmacological agent.

## Figures and Tables

**Table 1:** ** The regulation of key targets in *in-vivo* studies.** The effective level of target genes involved in BA synthesis and fatty acid uptake. The expression level of these genes is studied for FATP5 and the same has been evaluated against FXR, too.

Changes in Gene Expression	FXR (KO^*^)	FXR (CA)	FATP5 (KO)	References
*cyp7a1*	Up	Down	Up	(9,48,60,74)
SHP	Down	Up	Down	
NTCP	Up	Down	Up	
BSEP	Down	Up	Up	
OATP	Up	No change	No change	

**Table 2:** The steroidal and nonsteroidal agonist and antagonist for the bile acid receptors

Receptor	Target Tissue	Crystal information*	Steroidal	Activity	Non-Steroidal	Activity	References
FXR	Liver, intestine, kidney	Yes, 84*	CDCA > DCA > LCA > CA > UDCA, 5β-cholanoic acid, 5β-norcholanoic acid, 5α-cholanoic acid 6α-ethyl-CDCA, 6α-ethyl-3α,7α, 23-trihydroxy-24-nor-5β-cholan-23-sulphate (INT-767)	*Agonist* UDCA* (antagonist)* LCA *(antagonist)*	GW4064, Fexaramine, Way362450, PX-102,Cilofexor,Px-104,TERN-101, EYP001, Nidufexor, Tropifexor	*Agonist*	(7),(24),(13,68)
VDR	Intestine, kidney, bone	Yes, 49*	LCA, 3-keto-LCA	*Agonist*	LY2108491, LY2109886,vitamin D3 (Calcitriol) and Synthetic analogues (e.g. 2MbisP, BXL-01-0772)	*Agonist*	(7) ,(13),(75-77)
PXR	Liver, intestine	Yes, 23*	LCA, 3-keto-LCA >> CDCA, DCA, CA7α-hydroxy-4-cholesten-3-one	*Agonist*	Herbal medicine (e.g. Hyperforin, Guggulsterone), drugs (e.g. Rifampicin, Meclizine, Rifaximin Paclitaxel, Lovastatin	*Agonist*	(7),(13),(78-81)
CAR	Liver	Yes, 4*	CA, 6-keto-LCA, 12-keto-LCA	*Agonist*	Xenobiotic ligands: CITCO, TCPOBOP, Herbal medicine (e.g.6,7-dimethylesculetin), drugs (e.g. Phenobarbital.	*Agonist*	(7),(13),(80),(82)
TGR5	Liver, intestine, gallbladder, muscle, brain	No	LCA > DCA > CDCA > CA > UDCA TLCA BA analogs: INT-767, 6α-ethyl-23(S)-methyl-3α,7α,12α-trihydroxy-5β-cholan-24-oic acid (INT-777)	*Agonist*	Xenobiotic ligands: Oleanolic, Synthetic agonists: WB403, TRC210258,RDX8940	*Agonist*	(7),(13),(83-87)
S1PR2	Liver, intestine, Heart, brain	Yes, 3*	TCA, GCA, TCA, GDCA, TDCA, TUDCA	*Agonist*	NF	NF	(7),(88-90)
CHMR2	Heart	Yes, 9*	TCA	*Partial agonist*	NF	NF	(7),(91,92)
FATP5	Liver	No	DCA >UDCA>CDCA>CA	*Antagonist*	CB-2, CB-5, CB-6 and J3	*Antagonist*	(17), (34,93)

NF; not found; *Total number of crystal structures available in the Protein Data Bank for each bile acid receptors. >, higher affinity than; >>, much higher affinity than; TCA, taurocholic acid; GCA, glycocholic acid; GDCA, glycodeoxycholic acid; TUDCA, Tauroursodeoxycholic acid; CB-2((5E)-5-[(3-bromo-4-hydroxy-5-methoxyphenyl) methylene]-3-(3-chlorophenyl)-2thioxothiazolidin-4-one); CB-5, (2-benzyl-3-(4-chlorophenyl)-5-(4-nitrophenyl)-1H-pyrazolo[5,1-b] pyrimidin-7-one); CB-6(2-[7-(trifluoromethyl)-2,3-dihydro-1H-1,4-diazepin-5-yl] naphthalen-1-ol; J3,4-aryl-dihydropyrimidinones.

## Abbreviations

FXR: Farnesoid X receptor
NR: Nuclear receptor
FATP5: Fatty acid transport protein 5
TG: Triglyceride
ASBT: apical sodium-dependent bile salt transporter
BSEP: bile salt export pump
*cyp7a*1: cholesterol 7α-hydroxylase
ASBT: apical sodium-dependent bile salt transporter
BSEP: bile salt export pump
FGF19: fibroblast growth factor 19
FGFR4: fibroblast growth factor receptor 4
*SREBP-1c*: sterol regulatory element-binding protein 1c
NTCP: Na+-dependent taurocholate cotransport peptide
SHP: small heterodimer partner
BA-CoA: Bile acyl co-A
PXR: Pregnane X receptor
VDR: Vitamin D receptor
CAR: Constitutive androstane receptor
TGR5: Takeda G protein receptor 5
S1PR2: Sphingosine-1-phosphate receptor 2
CHRM2: Cholinergic receptor muscarinic 2
ACC: Acetyl CoA carboxylase
FAS: Fatty acid synthase
SCD1: stearyl CoA desaturase
ACS: Acyl Co-A synthetase
DAG: Diacylglycerol
GPAT: Glycerol-3-phosphate acyltransferases
PA: Lysophosphatidic acid
